# Outcome of ultrasound of the mammographically normal contralateral breast in patients recalled to the screening assessment clinic

**DOI:** 10.1186/bcr3785

**Published:** 2015-11-05

**Authors:** Preet Hamilton, Simon Lowes, Sheetal Sharma, Alicia Wright, Alice Leaver

**Affiliations:** 1 Gateshead Hospitals NHS Trust, Gateshead, UK

## Introduction

Women diagnosed with breast cancer are at increased risk of contralateral breast cancer; some of these cancers will be synchronous and mammographically occult (M1). Ultrasound may detect M1 breast cancers but also benign lesions that necessitate needle testing, conferring additional patient morbidity that could be termed 'over investigation'. Local guidelines for ultrasound of the M1 contralateral breast vary between units. We present a retrospective audit of contralateral M1 breast ultrasound within our screening assessment clinics.

## Methods

Screening and pathology hospital databases of 2013 and 2014 identified records of 331 women with screen-detected breast cancer. Descriptive statistics were performed.

## Results

All 331 women underwent ipsilateral breast ultrasound; 288 (87 %) underwent ultrasound of their contralateral mammographically normal (M1) breast. Six contralateral breast lesions were needle sampled: four B2 lesions, two B3 without atypia. No subsequent breast cancer has been detected in any of these patients to date.

## Conclusion

Two years of routine contralateral ultrasound has yielded no cancers but also very few benign biopsies. Ongoing audit and discussion of risk/benefit to patients is indicated.

